# Diagnostic Accuracy of a Handheld Ultrasound vs a Cart-based Model: A Randomized Clinical Trial

**DOI:** 10.5811/westjem.17822

**Published:** 2024-02-09

**Authors:** Ryan C. Gibbons, Daniel J. Jaeger, Matthew Berger, Mark Magee, Claire Shaffer, Thomas G. Costantino

**Affiliations:** * Lewis Katz School of Medicine at Temple University, Department of Emergency Medicine, Philadelphia, Pennsylvania; † Capital Health, Department of Emergency Medicine, Pennington, New Jersey; ‡ University of Pittsburgh Medical Center Harrisburg, Department of Emergency Medicine, Harrisburg, Pennsylvania

## Abstract

**Introduction:**

Numerous studies have demonstrated the accuracy of point-of-care ultrasound (POCUS). Portable, handheld devices have expanded the clinical scope of POCUS at a fraction of the cost of traditional, cart-based models. There is a paucity of data assessing the diagnostic accuracy of portable devices. Our objective in this study was to compare the diagnostic accuracy of a portable device with a cart-based model.

**Methods:**

This was an institutional review board-approved, observational, prospective, randomized clinical trial (NCT05196776) of a convenience sample of adult patients who presented to a university-based health system. Patients who required a cardiac, lung, renal, aorta, or biliary POCUS were randomized to a portable device or to a cart-based model. We hypothesized that the cart-based model would have a 90% diagnostic accuracy vs 70% for the handheld device. To detect a 20% difference, the sample size was calculated to be 98, with 49 patients randomized to each arm. We used standard 2x2 tables to calculate test characteristics with 95% confidence intervals (CI).

**Results:**

A total of 110 patients were enrolled, with 56 patients randomized to the cart-based model and 54 to the handheld device. The sensitivity, specificity, and diagnostic accuracy of the cart-based vs handheld were 77.8% (40–97.2) vs 92.9% (66.1–99.8), 91.5% (79.6–97.6) vs 92.3% (79.1–98.4%), and 89.3% (78.1–96) vs 92.5% (81.8–97.9), respectively.

**Conclusion:**

The diagnostic accuracy of a portable, handheld device is similar to that of a cart-based model.

Population Health Research Capsule What do we already know about this issue?

*Point-of-care ultrasound (POCUS) enhances our ability to safely, efficiently, and accurately diagnose and manage our patients.*

What was the research question?

*Does a handheld POCUS device have similar diagnostic accuracy as a traditional, cart-based model?*

What was the major finding of the study?

*A handheld POCUS device has similar diagnostic accuracy as a traditional, cart-based model (sensitivity 77.8% vs. 92.9%, specificity 91.5% vs. 92.3% and accuracy 89.3% vs. 92.5%).*

How does this improve population health?

*Given the similar diagnostic accuracies, handheld devices broaden the availability of POCUS and enhance patient care in resource-limited settings.*

## INTRODUCTION

Numerous studies have demonstrated the accuracy of point-of-care ultrasound (POCUS) to diagnosis pathology and to augment procedural guidance.^1^ ^–^ ^10^ Portable, handheld devices have expanded the clinical scope of POCUS across diverse settings, including prehospital, resource-limited, and outpatient clinics.^11^ ^–^ ^13^ The majority of existing literature has assessed the timeliness and image quality of handheld devices only.^13^ ^–^ ^15^ To date, there is a paucity of data assessing the diagnostic accuracy of these portable devices.^16^ ^–^ ^24^ To our knowledge, there are no randomized studies comparing the diagnostic accuracy of a portable, handheld device with a traditional cart-based model. Our primary objective in this study was to compare the diagnostic accuracy of these two diagnostic imaging modalities, specifically for cardiac, lung, biliary, renal and abdominal aorta imaging, Secondary analysis included assessment of image quality.

## METHODS

### Study Design

This was an institutional review board-approved, observational, prospective, randomized clinical trial (NCT05196776) with parallel assignment and an allocation ratio of 1∶1. We followed the CONSORT guidelines and checklists for clinical trials. Butterfly Network, Inc. provided funding for this study.

### Study Setting and Population

Between October 1–December 31, 2021 we included a convenience sample of patients ≥18 years old, who presented to one of three clinically distinct emergency departments (ED) affiliated with an urban, Level I, university-based health system with >200,000 adult and pediatric visits annually, and who required a cardiac, lung, biliary, renal, or abdominal aorta POCUS based on the discretion of the emergency attending physician (EP). Study investigators enrolled patients capable of providing written informed consent. Our department credentials all EPs in the core POCUS applications as defined by the American College of Emergency Physicians (ACEP).^25^ All English- and Spanish-speaking patients requiring a POCUS evaluation were eligible for enrollment. We excluded patients unable to consent.

### Study Protocol

We used permuted-block randomization with an allocation ratio of 1∶1. Allocation concealment included sequentially numbered, opaque, sealed envelopes. Upon enrollment, blinded study investigators selected an envelope containing study materials and pre-randomized selection into the handheld device (HH) or cart-based model (CB) using Research Randomizer version 4.0 (www.randomizer.org).^26^ Patients, who required a cardiac, lung, renal, aorta, or biliary POCUS, were randomized to a portable device, the Butterfly iQ (Butterfly Network, Inc, Guilford, CT) transducer connected to a fifth generation Apple iPad Mini (Apple Inc, Cupertino, CA), or to a cart-based model, the GE Venue Go or GE Logiq E (GE HealthCare, Wauwatosa, WI). (Refer to Image.) We studied the five most commonly performed POCUS scans in our department.

**Image.**
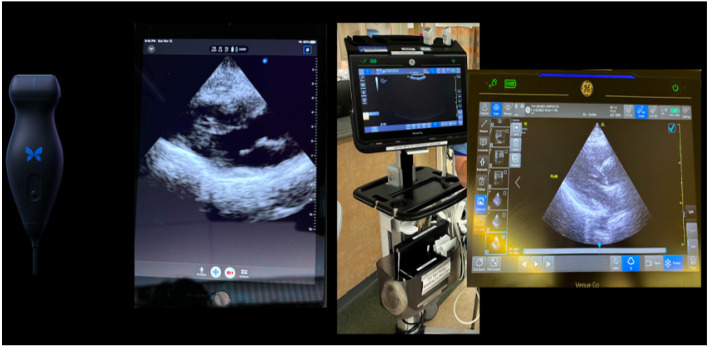
Handheld Butterfly iQ device and cart-based GE Venue Go model demonstrating parasternal long axis view.

Using the phased array transducer (2–5 mHz) for cardiac imaging or the curvilinear transducer (1-mHz) for the lung, renal, aortic, and biliary scans, postgraduate year 1–3 emergency medicine (EM) residents performed each POCUS prior to advanced imaging. Performing physicians used the corresponding settings for the HH device. An attending EP, credentialed in the core ACEP POCUS applications, reviewed each study concurrently. Study investigators blinded all residents performing the scans and the attending EPs reviewing them to the study objective and its funding.

A cardiologist-interpreted echocardiogram, performed within 24 hours of presentation to the ED, served as the reference standard for cardiac images. For biliary tract images, the reference standard was a radiology-interpreted ultrasound performed during the ED visit. For lung, renal and aortic scans, the reference standard was computed tomography images (when available and performed during the ED visit), or POCUS quality assurance (QA) review by two ultrasound fellowship-trained physicians (when no CT was available). If there was disagreement, a third ultrasound fellowship-trained physician provided an interpretation. The cardiologist, radiologist, and the ultrasound fellowship-trained EPs were blinded to the real-time POCUS reads. However, the EPs performing QA knew about the study and its funding.

Prior to starting their internship, our EM residents participate in an introductory five-hour Introduction to POCUS course taught by our emergency ultrasound faculty. Additionally, each resident completes a three-week emergency ultrasound rotation during their internship in accordance with Accreditation Council for Graduate Medical Education (ACGME) and ACEP guidelines.^24^ ^,^ ^27^ Residents received no additional training prior to their study participation. Nonetheless, each participant completed more than 25 of each scan prior to participating in the study to achieve competency per ACEP and AGME guidelines.^24^ ^,^ ^27^

### Measurements

Prior to study commencement, we defined the following diagnostic endpoints: ejection fraction (EF) (good >50%, moderate 30–50%, poor <30%) and the presence or absence of the following: gallstones; hydronephrosis (mild, moderate, or severe); abdominal aortic aneurysm (>3 centimeters), and B-lines (≥3 in a single lung field or a single, confluent B-line occupying >1/3 of the intercostal window).^28^ The presence of B-lines indicates an interstitial process, whether localized or diffuse, reflects its etiology. We compared this to interstitial findings on CT (if available) of the corresponding lobe. We did not compare additional measurements (ie, gallbladder wall thickness, or assess M-mode or Doppler findings). The study included B-mode findings only. Using the electronic health record (Epic Systems Corp, Verona, WI), we performed chart abstraction on all patients to obtain results of cardiology-interpreted echocardiograms and radiology-interpreted ultrasound and CT studies.

Diagnostic accuracy of each imaging modality compared to the aforementioned gold standards served as the primary endpoint. Image quality served as the secondary endpoint. Three ultrasound fellowship-trained physicians used a previously validated Likert scale to assess image quality.^29^ A score of 1 indicated unable to interpret, and a score of 7 specified superior imaging quality.

### Statistical Analysis

Prior studies assessing POCUS performed using traditional CB technology have demonstrated the following sensitivities for respective pathologies: EF (89%); cholelithiasis (94%); abdominal aortic aneurysms (97%); B-lines (92%); and hydronephrosis (75%), providing an average sensitivity of 90%.^28^ ^,^ ^30^ ^–^ ^37^ Given the lack of pre-existing data comparing the modalities, we hypothesized that the HH device would have an overall sensitivity of 70%. We postulated that the HH would be inferior given the smaller screen size, novel technology to generate sonographic images, and limited clinician experience with the device. Based on a power of 80% and an alpha of 0.05, we calculated a sample size of 98, with 49 patients randomized to each arm, to detect a 20% difference. We report continuous and categorical data as medians with interquartile ranges (IQR) or proportions with 95% confidence intervals (CI), and we used standard 2 × 2 tables to calculate test characteristics with 95% CIs using MedCalc version 19.1.6 (MedCalc Software Ltd, Ostend, Belgium). Intraclass correlation coefficient assessed inter-rater reliability between blinded expert reviewers, and we used the *t*-test to compare median Likert scores.

## RESULTS

**Figure 1.**
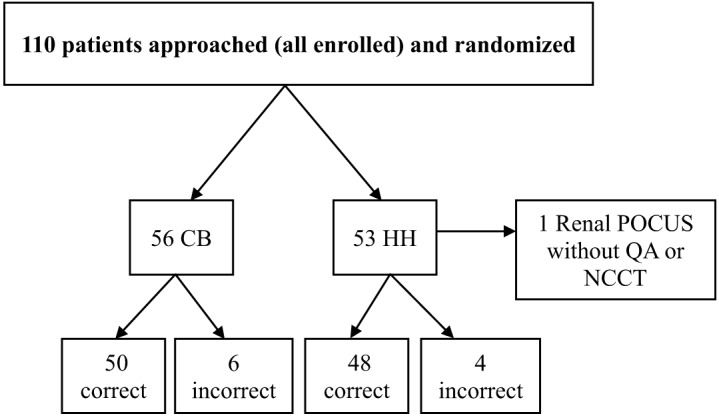
Patient flow chart. *CB*, cart-based ultrasound model; *HH*, handheld device; *POCUS*, point-of-care ultrasound; *QA*, quality assurance review; *NCCT*, non-contrast computed tomography of the abdomen and pelvis.

We enrolled 110 patients with 56 patients randomized to the CB model and 54 to the HH device (Figure 1). Authors excluded one HH patient given there were no sonographic images available to review. Table 1 illustrates the similarity of patient characteristics and the number of each POCUS type across both cohorts (Table 1). Table 2 portrays test characteristics for each diagnostic modality, while Tables 3 and 4 depict the diagnostic criterion reference used and the diagnostic inaccuracies, respectively.

**Table 1.** Patient characteristics.

Characteristic	Cart-based model (n = 56)	Handheld device (n = 53)
Age, median (IQR), years	57 (18–90)	60 (18–89)
Gender, N (%)		
Female	60.7	51
Male	39.3	49
Body mass index, median (IQR)	30 (22–64.9)	27.9 (15–42.2)
Point-of-care ultrasound scans	Aorta (9)	Aorta (4)
	Cardiac (11)	Cardiac (17)
	Gallbladder (10)	Gallbladder (14)
	Lung (11)	Lung (7)
	Renal (15)	Renal (11)

*IQR*, interquartile range.

**Table 2.** Test characteristics.

	Cart-based model (n = 56; 95% CI)	Handheld device (n = 53; 95% CI)
Sensitivity	77.8 (40–97.2)	92.9 (66.1–99.8)
Specificity	91.5 (79.6–97.6)	92.3 (79.1–98.4)
Positive likelihood ratio	9.2 (3.4–24.9)	12.1 (4.0–36.2)
Negative likelihood ratio	0.2 (0.1–0.8)	0.1 (0–0.5)
Disease prevalence	0.2	0.3
Positive predictive value	63.6 (39.2–82.6)	81.3 (59.1–92.9)
Negative predictive value	95.6 (86.3–98.7)	97.3 (84.5–99.5)
Accuracy	89.3 (78.1–96)	92.5 (79.3–96.9)

*CI*, confidence interval.

**Table 3.** Diagnostic criterion reference used for comparison.

	Cart-based model (n = 56)		Handheld device (n = 53)	
	QA	Echo	QA	Echo
Cardiac (n = 27)	5	6	8	8
	QA	RUQ US	QA	RUQ US
Biliary (n = 24)	10	0	10	4
	QA	CT	QA	CT
Lung (n = 18)	11	0	5	2
Renal (n = 27)	13	2	9	3
Aorta (n = 13)	6	3	0	4

*QA*, quality assurance review; *Echo*, cardiology-performed and interpreted echocardiography; *RUQ US*, radiology- performed and interpreted right upper quadrant ultrasound; *CT*, computed tomography of the chest or abdomen and pelvis with or without contrast.

**Table 4.** Diagnostic inaccuracies by imaging modality.

	Cart-based model (n = 6)	Handheld device (n = 4)
Cardiac (n = 5)	2 interpreted as normal EF, read as moderate during QA	1 interpreted as normal EF, read as moderate during QA 1 interpreted as moderate EF, read as normal during QA 1 interpreted as poor EF, read as normal on echo
Biliary (n = 0)	0	0
Lung (n = 0)	0	0
Renal (n = 4)	2 interpreted as mild hydronephrosis, read as normal during QA 1 interpreted as moderate hydronephrosis, read as normal during QA 1 interpreted as moderate hydronephrosis, read as normal on NCCT	1 interpreted as mild hydronephrosis, read as normal on NCCT
Aorta (n = 0)	0	0

*EF*, ejection fraction; *QA*, quality assurance review; *Echo*, cardiology performed and interpreted echocardiography; *RUQ US*, radiology performed- and interpreted right upper quadrant ultrasound; *NCCT*, non-contrast computed tomography of the abdomen and pelvis; *CB*, cart-based model; *HH*, handheld device.

Overall, there were 10 incorrect diagnoses, four for the HH device and six for the CB model. Table 4 highlights the diagnostic inaccuracies by scan type, diagnostic modality, and criterion reference. The HH correctly identified the following: six instances of cholelithiasis; one case of mild and one of moderate hydronephrosis; four individuals with pulmonary edema; and one patient with a moderate EF. The CB modality correctly identified the following: two instances of cholelithiasis; one case of severe hydronephrosis; two individuals with pulmonary edema; and two patients with poor EFs. The median Likert score for CB was 5, and 4 for the HH. Intraclass correlation coefficients for the HH and CB were 0.5 (95% CI 0.2–0.7) and 0.8 (95% CI 0.7–0.8), respectively.

## DISCUSSION

To the best of our knowledge, ours is the first published randomized trial comparing a portable HH device with a traditional CB model in ED patients. Given the lack of pre-existing data, we hypothesized that the traditional CB model would be superior with respect to diagnostic accuracy and image quality. Handheld devices are still novel and have not been adopted broadly, limiting clinician experience. Moreover, novel technology to generate sonographic images, compared to the traditional piezoelectric crystals, may affect image quality as well. Similarly, we assumed screen resolution and size would limit image quality and, subsequently, accuracy. However, a small pilot study by Magee et al demonstrated similar results between HH and CB devices when interpretating pre-recorded videos assessing for free fluid in the right upper quadrant.^13^

We chose five basic POCUS examinations that our EPs have considerable experience performing with appropriate diagnostic accuracy. Our EPs currently have less experience with other POCUS indications, such as regional anesthesia and fracture assessment. Moreover, we did not have access to a HH endocavitary transducer to assess for pregnancy-related issues. These areas are ripe for future research. Overall, we found no significant difference in sensitivity or specificity between CB and HH ultrasound images. However, this limited our sample size for each modality.

Although the study types and indications varied, the idea of diagnostic accuracy should apply to all POCUS studies. It is probably expected that when the diagnosis was the objective presence or absence of a finding, (ie, gallstones) there were no misdiagnoses.^29^ ^,^ ^30^ However, when the diagnosis was more subjective (ie, estimating EF or the degree of hydronephrosis) there were more inaccuracies across both modalities. This is consistent with previous studies showing more overlap of good and moderate EFs and between poor and moderate.^31^ In our study, there was a tendency to overestimate the presence or degree of hydronephrosis, which is likely confirmational bias in the setting of a presumed nephrolithiasis diagnoses.

As expected, the CB device had better overall image quality than the HH. However, this did not affect diagnostic accuracy, as our results suggest that it is similar between HH and CB modalities in an academic EM residency. Superior image quality may detect more subtle pathology, such as signs of cholecystitis.^32^ Each diagnostic modality serves a clinical role. This data can be extrapolated to the broader EM community with the increasing prevalence of ultrasound competency in practicing EPs and availability of portable devices. Furthermore, it supports the utility of HH devices in resource-limited settings, outpatient clinics, and inpatient locations with limited access to traditional sonographic machines, not to mention pandemic settings where disinfection is paramount.^2^

## LIMITATIONS

This study suffers from the limitations of an observational design with convenience sampling at a single health system resulting in a selection bias as well as a smaller sample size, which limits the level of precision to exclude a type II error. Using the discretion of the attending EP to determine whether a patient needed a specific POCUS examination created a selection bias as well. We did not define specific indications to perform one of the aforementioned POCUS scans. Moreover, we hypothesized the diagnostic accuracy of the HH device given the lack of pre-existing data. This limits the validity of our power analysis.

Butterfly Network, Inc. funded the study, which may have introduced bias. However, physicians performing the ultrasounds were unaware of this funding. Furthermore, physicians performing the ultrasound had significantly more experience using the CB model compared to the HH device, which may have introduced bias in favor of the traditional modality. Furthermore, we did not account for the experience level of the residents performing the ultrasound, which could have impacted quality and accuracy. Presumably, senior residents had more proficiency.

We did not compare additional types of HH devices. Therefore, it is unclear whether our data is applicable to other devices using different technology. Specifically, the Butterfly iQ device uses chip technology compared to traditional piezoelectric crystals. This may impact image quality and diagnostic accuracy. Presumably, the HH frequencies settings for each study reflect those of the traditional CB modalities. However, we did not account for software features, screen size, or resolution in our study. Future studies need to validate our findings across the array of HH devices and emerging technology. Furthermore, we limited our study to only five of the ACGME core ultrasound competencies. Therefore, additional studies are needed to validate our findings to broader POCUS applications, including various settings such as M-mode and Doppler.

Using the subjective interpretation of ultrasound fellowship-trained faculty as the criterion reference when other standard diagnostics imaging modalities were not done limits the validity of the results and causes a misclassification bias. Specifically, we did not account for the potential for inferior technology. For example, if the HH or CB model provides inferior imaging, not only may the performing physician miss pathology, but the EPs conducting QA may overlook it as well. This false negative may not be missed by a radiology-performed and interpreted ultrasound. Moreover, reviewers were not blinded to the image source, HH vs CB, given that each modality uses unique storage means. Nonetheless, quality assurance review is common practice in academic EDs with an ultrasound division, and confirmatory studies are typically unnecessary.

Additionally, using cardiologist-obtained echocardiograms as a reference standard introduces the potential for treatment effects between when the POCUS images were obtained and when the cardiology images were obtained. While each patient received a cardiology echocardiogram within 24 hours of the ED visit to limit such effects, this is nonetheless a limitation to our study. Finally, our ED is not representative of the broader EM community. We have an active ultrasound division with numerous faculty and fellows. All EPs are credentialed in POCUS. In our department, residents are the treating clinicians, who typically have more POCUS experience compared to most practicing EPs. Furthermore, our department has regular access to and experience with portable devices.

## CONCLUSION

The diagnostic accuracy of a portable, handheld ultrasound device is similar to the accuracy of a traditional, cart-based model when performing cardiac, lung, biliary, renal, or abdominal aorta studies. Future larger, multicenter studies are required to validate these findings.
